# An Extension of the Stern–Volmer Equation for Thermally Activated Delayed Fluorescence (TADF) Photocatalysts

**DOI:** 10.1021/acs.jpclett.4c02609

**Published:** 2024-10-11

**Authors:** Bart Limburg

**Affiliations:** † Secció de Química Orgànica, Facultat de Química, Universitat de Barcelona, Carrer Martí i Franquès 1-11, 08028 Barcelona, Spain; ‡ Institut de Química Teòrica i Computacional (IQTC), Carrer Martí i Franquès 1-11, 08028 Barcelona, Spain

## Abstract

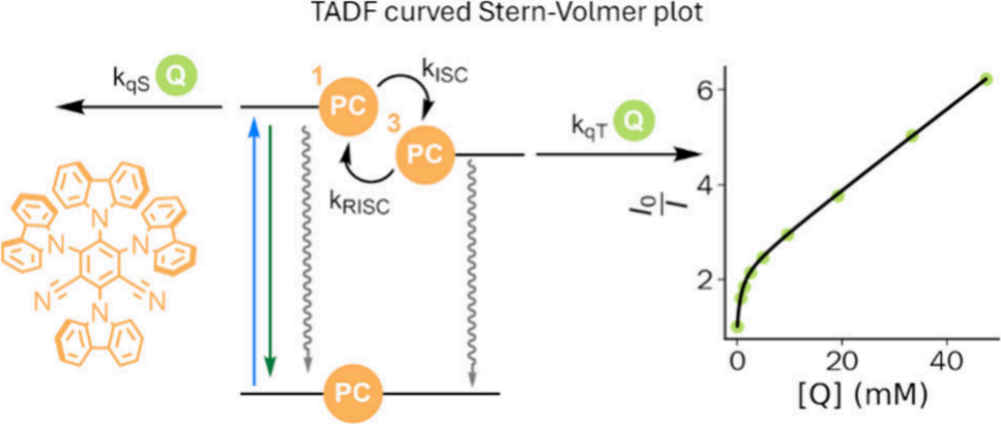

Fluorescence quenching experiments are essential mechanistic tools in photoredox catalysis, allowing one to elucidate the first step in the catalytic cycle that occurs after photon absorption. Thermally activated delayed fluorescence (TADF) photocatalysts, however, yield nonlinear Stern–Volmer plots, thus requiring an adjustment to this widely used method to determine the efficiency of excited state quenching. Here, we derive an extension of the Stern–Volmer equation for TADF fluorophores that considers quenching from both the singlet and triplet excited states and experimentally verify it with fluorescence quenching experiments using the commonly employed TADF-photocatalyst 4CzIPN, and multiple-resonance TADF-photocatalyst QAO with three different quenchers in four solvents. The experimental data are perfectly described by this new equation, which in addition to the Stern–Volmer quenching constants allows for the determination of the product of intersystem and reverse intersystem crossing quantum yields, a quantity that is independent of the quencher.

The family of thermally activated delayed fluorescence (TADF) dyes based on diarylamino-functionalized dicyanobenzenes^1^ has been extensively applied in fields such as organic electronics and photoredox catalysis during the past decade. In the latter field, light excitation of the TADF dye leads to a reactive excited state that can be quenched through electron or energy transfer, leading to reactivity that is otherwise absent in the ground state.^2^ Photocatalysts based on transition-metal complexes of, e.g., Ir or Ru,^3^ or non-TADF organic dyes^4^ feature emission from a pure excited state. In most cases, the reactive excited state is a triplet that occurs after absorption of light and intersystem crossing (ISC), because triplet states feature longer lifetimes that allow for bimolecular reactivity. TADF dyes, however, are characterized by an excited-state system that, in its simplest form, has two excited states (a singlet and a triplet) that are close enough in energy such that, in addition to ISC, thermal population of the higher-energy singlet excited state from the triplet, known as reverse intersystem crossing (RISC), occurs readily at room temperature.^2^ After excitation, these compounds decay back to the ground state in a biexponential manner according to eq 1 which can be interpreted as a *prompt* decay of the initially pure singlet excited state (with rate constant *k*_p_), and a slower delayed decay of an equilibrium state of singlet and triplet excited states (with rate constant *k*_d_); see Figure 1a.
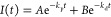
1It is important to note that the experimentally observed rate constants of this biexponential decay (*k*_p_ and *k*_d_) are not the inverse of the lifetime of the pure singlet and triplet states but instead are mathematically defined as follows.^5^
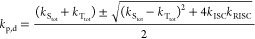
2Here *k*_S_tot__ and *k*_T_tot__ are defined as the sum of all rate constants of the *unimolecular* processes (i.e., not including the bimolecular processes discussed in the following sections) occurring from the singlet and triplet state, respectively. *k*_S_tot__ and *k*_T_tot__, as well as the product of the rate constants of ISC and RISC (*k*_ISC_*k*_RISC_) can be extracted by considering the ratio of fluorescence intensity between the two individual decays as measured by time-correlated single photon counting, according to eqs 3–5 (see the Supporting Information).^5^ This ratio should be measured with sufficient data points on the prompt decay to ensure the ratio is reliable, and therefore it is best to do one measurement that captures the entire decay and one that mostly captures the prompt decay; see the Supporting Information.
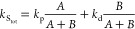
3
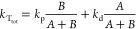
4
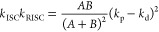
5

**Figure 1:**
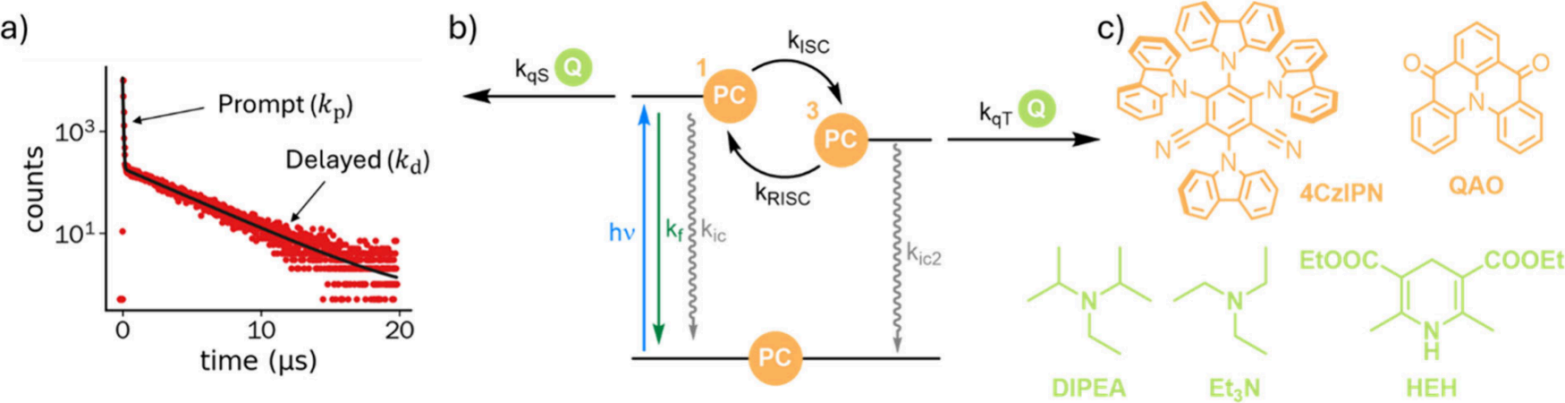
a) TCSPC data (red circles) of 4CzIPN (50 μM in THF) at 20 °C, λ_ex_ = 446 nm, showing a biexponential decay, and corresponding fit to eq 1 (black line). b) Simplified Jablonski diagram of TADF fluorophores and possible bimolecular quenching pathways from either the singlet or triplet excited states. c) Molecular structure of TADF-fluorophore 4CzIPN, MR-TADF-fluorophore QAO, and the quenchers employed in this study: triethylamine (Et_3_N), diisopropylamine (DIPEA), and Hantzsch Ester (HEH).

In the field of photoredox catalysis, it is important to understand which compound in the complex reaction mixture reacts with the excited state of the photocatalyst, and for this reason so-called luminescence quenching experiments are often an integral part of any mechanistic work.^6−14^ In such studies, the luminescence intensity of the photocatalyst is probed under varying concentrations of the quencher compound, and the results are plotted using the Stern–Volmer plot, generally obtaining a linear relationship for well-behaved dynamically quenched systems when utilizing photocatalysts with a single (emissive) excited state, according to eq 6.
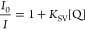
6

Many physical and chemical effects, however, can lead to a deviation from linearity such as static quenching,^15,16^ high viscosity,^17^ and inner-filter effects.^9,16,18^ Interestingly, TADF photocatalysts also exhibit nonlinear Stern–Volmer plots portraying a negative curvature.^6,19^ Within the photoredox catalysis community, however, these fluorophores are typically treated as if they were photocatalysts with a single excited emissive state, which can lead to incorrect interpretations of the luminescence quenching studies. In this work, we derive and experimentally verify an extension of the Stern–Volmer equation that fully takes into account the rapid singlet–triplet interconversions that define the excited state of TADF photocatalysts and considers quenching from both the singlet and triplet excited state (Figure 1b).

We first consider the quantum yield of fluorescence since it is proportional to the luminescence intensity *I*. The description of the quantum yield of fluorescence in the absence of bimolecular reactions for TADF fluorophores (Φ_f_^0^) needs to consider the prompt as well as the delayed components and take into account that the system can go through several cycles of ISC and RISC before ultimately decaying to the ground state. Taking the limit of the resulting geometric series (eq 7) yields the eq 8.^20^
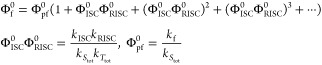
7
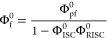
8Here, Φ_pf_^0^ is the quantum yield of prompt fluorescence, and Φ_ISC_^0^ and Φ_RISC_^0^ are the quantum yields of intersystem crossing and reverse intersystem crossing, respectively. Having defined the quantum yield of fluorescence, we can consider bimolecular reactions occurring from both the singlet and the triplet excited states (Figure 1b). The mathematical description of such reactions is identical for electron transfer and energy transfer reactions. Each possible reaction has its corresponding rate constant and depends on the concentration of the quenching compound, i.e., *k*_qS_[Q] or *k*_qT_[Q] for reactions of compound Q with the singlet or triplet excited state, respectively. The quantum yields defined above are all affected by these reactions, and their definitions in the presence of quencher Q are as follows:
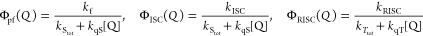
9

Since the ratio of the emission intensities is equal to the ratio of fluorescence quantum yields, we divide Φ_f_^0^ by Φ_f_(*Q*) to derive the extension of the Stern–Volmer equation (see the Supporting Information):
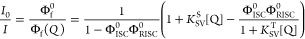
10Here, *K*_SV_^S^ and *K*_SV_^T^ are the Stern–Volmer constants for quenching the singlet and triplet excited states:  and .

Equation 10 was experimentally verified by employing the common TADF photocatalyst 4CzIPN, and three different quenchers: triethylamine (Et_3_N), diisopropylethylamine (DIPEA), and Hantzsch Ester (HEH) in four different solvents (Figure 2; see Figure 1c for chemical structures). The fluorescence quenching was measured in strictly deoxygenated conditions (at least three freeze–pump–thaw cycles) at various concentrations of quencher, and the three-parameter eq 10 was used to fit the experimental data as shown in Figure 2. The resulting fitted constants are listed in Table 1. The data in Figure 2 clearly deviate from the typical linear relationship of the Stern–Volmer equation (eq 6), which can be rationalized considering the presence of two excited states where it is easier to quench the triplet state than the singlet state due to the much longer lifetime of the former. As such, efficient triplet-state quenching leads to a considerably larger Stern–Volmer constant for the triplet state *K*_SV_^T^ than for the singlet state *K*_SV_^S^ (cf. values in Table 1). Upon increasing the concentration of quencher, initially, the quenching is very effective followed by more moderate quenching. After the initial quenching the triplet state is completely deactivated (i.e., RISC is outcompeted by quenching, and the term  becomes 0), after which the TADF photocatalyst behaves as if it were a fluorophore comprising only a singlet excited state, and the plot becomes linear. Literature reports of fluorescence quenching experiments using TADF fluorophores indeed often depict linear Stern–Volmer plots, which can occur when the first data point is taken at a concentration at which full triplet quenching already occurs. However, in such cases, the *y*-intersect (i.e., [Q] = 0) is at a value greater than 1 (see Figure 3a), which is an easily overlooked indication that eq 6 does not apply. Instead, in the hypothetical limit where  is 0, eq 10 predicts that the *y*-axis intersect of an (inaccurate) linear fit occurs at . In addition, the slope of the linear part of the plot does not have a slope of *K*_SV_^S^, but instead *K*_SV_^S^/(1 – Φ_ISC_^0^Φ_RISC_^0^), therefore overestimating the amount of singlet-state quenching if the data was analyzed using eq 6 instead. For the same reason, linear plots might be obtained if dioxygen exclusion is insufficiently performed, which likewise does not yield accurate fitted constants.

**Figure 2:**
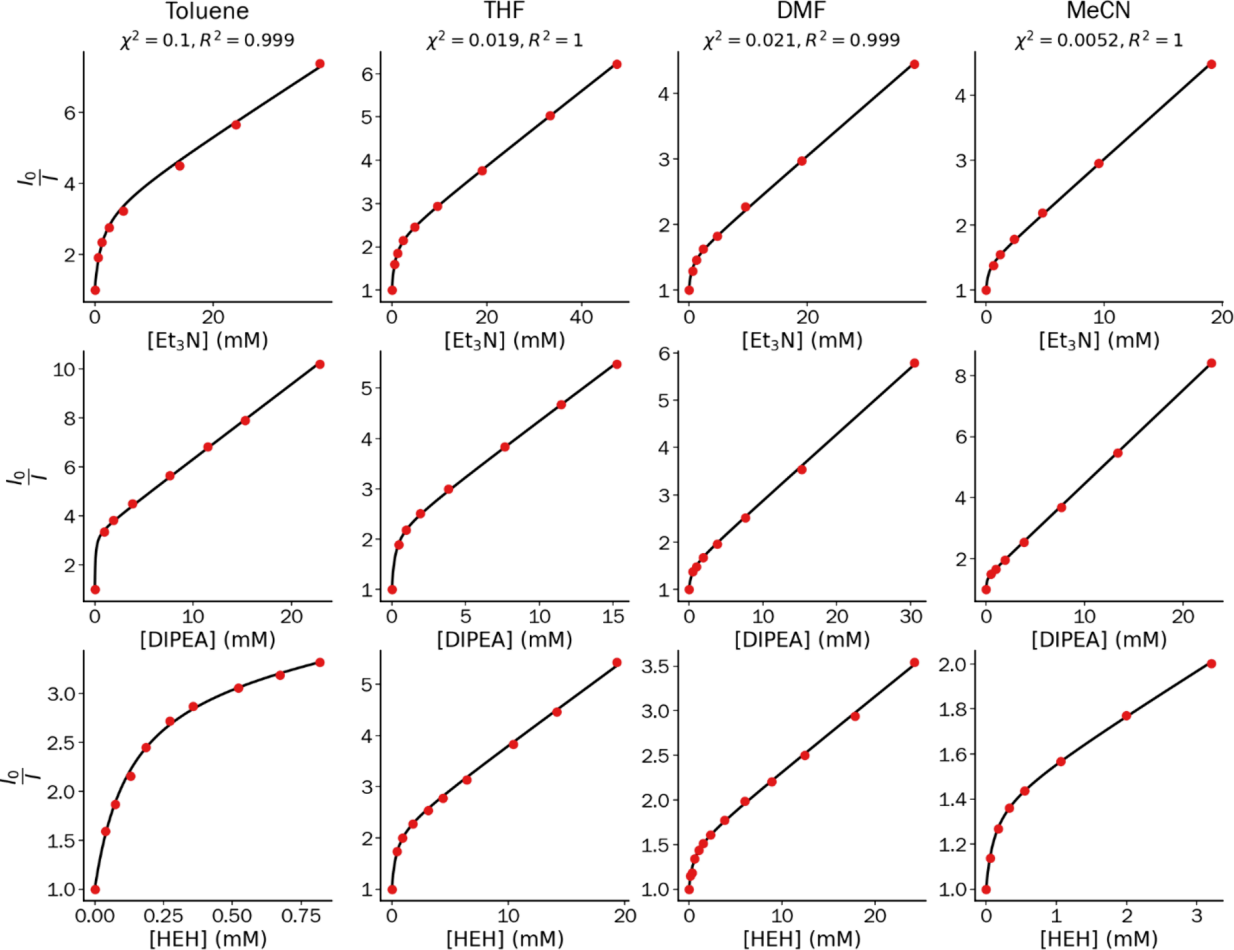
Stern–Volmer plots of TADF-photocatalyst 4CzIPN (50 μM) in four solvents (toluene, THF, DMF, and MeCN) with three different quenchers (Et_3_N, DIPEA, HEH) thermostated at 20 °C, *λ*_ex_ = 450 nm. Global fits (taken for all three quenchers per solvent) to eq 10 are shown as the black continuous line, and goodness of fit χ^2^ and *R*^2^ are indicated for each global fit.

**Table 1:** Obtained Fitting Constants from Fluorescence Quenching Experiments in Figure 2 and TCSPC (See the Supporting Information)^a^

Solvent	Quencher	*K*_SV_^S^ (M^–1^)^b^	*K*_SV_^S^ × 10^3^ (M^–1^)^b^	Φ_ISC_^0^ Φ_RISC_^0^^b^	*k*_S_tot__ × 10^7^ (s^–1^)^c^	*k*_T_tot__ × 10^5^ (s^–1^)^c^	*k*_qS_ × 10^9^ (M^–1^ s^–1^)	*k*_qT_ × 10^9^ (M^–1^ s^–1^)
Toluene	Et_3_N	32 ± 1.4	0.855 ± 0.09	0.70 ± 0.006	6.68 ± 0.97	8.73 ± 0.75	2.2 ± 0.33	0.75 ± 0.10
	DIPEA	93 ± 3.3	13.9 ± 0.98	(0.71 ± 0.17)^c^			6.2 ± 0.93	12 ± 0.87
	HEH	125 ± 33	8.00 ± 0.70				8.4 ± 2.5	7.0 ± 0.86
THF	Et_3_N	39 ± 0.69	1.48 ± 0.13	0.54 ± 0.005	3.65 ± 0.55	6.50 ± 0.58	1.4 ± 0.21	0.96 ± 0.12
	DIPEA	100 ± 1.9	4.70 ± 0.59	(0.58 ± 0.15)^c^			3.7 ± 0.55	3.1 ± 0.47
	HEH	76 ± 1.5	2.67 ± 0.26				2.8 ± 0.42	1.7 ± 0.23
DMF	Et_3_N	53 ± 1.2	2.25 ± 0.52	0.33 ± 0.009	3.96 ± 0.40	8.44 ± 0.58	2.1 ± 0.22	1.9 ± 0.46
	DIPEA	94 ± 1.8	2.87 ± 0.66	(0.35 ± 0.06)^c^			3.7 ± 0.38	2.4 ± 0.58
	HEH	57 ± 1.5	2.20 ± 0.37				2.2 ± 0.23	1.9 ± 0.33
MeCN	Et_3_N	116 ± 1.8	5.15 ± 1.4	0.29 ± 0.006	5.13 ± 0.64	8.26 ± 0.74	6.0 ± 0.75	4.3 ± 1.2
	DIPEA	218 ± 2.6	14.9 ± 7.5	(0.30 ± 0.06)^c^			11 ± 1.4	12 ± 6.3
	HEH	139 ± 5.6	8.56 ± 1.2				7.1 ± 0.93	7.1 ± 1.2

a Uncertainties are the (propagated) standard errors of the fitted constants.

b Obtained through fluorescence quenching experiments.

c Obtained from fitting the time-correlated single photon counting decays.

**Figure 3:**
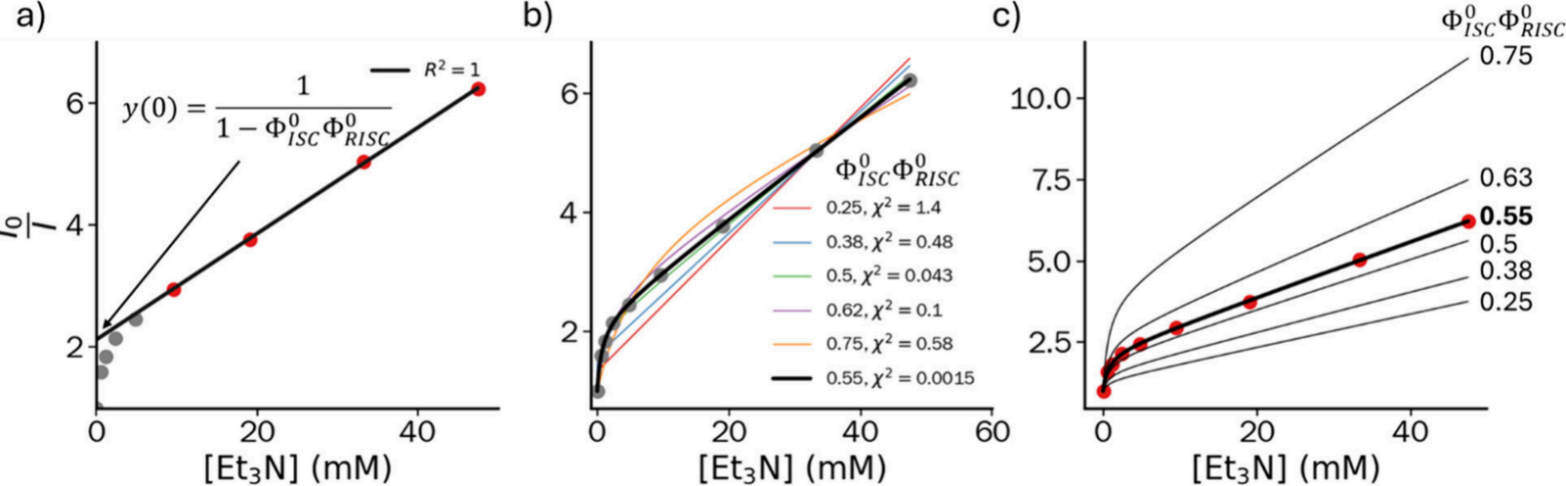
Quenching experiment of 4CzIPN (50 μM) with Et_3_N in THF at 20 °C and various fits. a) Linear fit using only the higher-concentration data points (red), discarding the data at lower concentration (gray). b) Fits using fixed values of Φ_ISC_^0^Φ_RISC_^0^ demonstrating that it is not possible to fit the data (circles) fixing different values for Φ_ISC_^0^Φ_RISC_^0^ (cf. χ^2^ values), highlighting that the equation is not overparametrized. Only when Φ_ISC_^0^Φ_RISC_^0^ is allowed to vary is a good fit obtained (bold black line). c) Simulations using different values for Φ_ISC_^0^Φ_RISC_^0^, using fitted values for *K*_SV_^S^ (39 M^–1^) and *K*_SV_^T^ (1480 M^–1^) obtained from the bold line fit to the data (circles).

For each solvent, the data obtained for the three different quenchers can be fitted with the same value of Φ_ISC_^0^Φ_RISC_^0^ (in Figure 2, a global fit was performed with Φ_ISC_^0^Φ_RISC_^0^ as a shared parameter between the data of the three quenchers in the same solvent; see the Supporting Information for individual fits per quencher). This is expected as this constant is a property of the fluorophore (in a certain solvent) and does not depend on the quencher. In contrast, the fitted value for Φ_ISC_^0^Φ_RISC_^0^ does differ when changing the solvent due to the dependence of the unimolecular rate constants (e.g., *k*_ISC_ and *k*_RISC_) on the solvent polarity.^21^ The value for Φ_ISC_^0^Φ_RISC_^0^ obtained through fluorescence quenching experiments closely matches that from fitting the TCSPC data (see Table 1), further confirming that eq 10 describes well the experimental data and that therefore intrinsic properties of the TADF fluorophore can be obtained by simple steady-state fluorescence quenching experiments. Additionally, the precise value of the property Φ_ISC_^0^Φ_RISC_^0^ can be obtained to greater accuracy using quenching experiments than through TCSPC experiments by performing global fitting on data sets with more than one quencher. Using a combination of steady-state fluorescence quenching and a single TCSPC experiment in the absence of quencher, the quenching rate constants *k*_qS_ and *k*_qT_ can be determined, since *k*_S_tot__ and *k*_T_tot__ can be obtained from the biexponential fit of the TCSPC decay (*vide supra*). Table 1 lists the quenching rate constants, showing that quenching of both the S_1_ and T_1_ state of 4CzIPN is very efficient and close to the diffusion limit, with only small variation between the two, even though the Stern–Volmer constants *K*_SV_^S^ and *K*_SV_^T^ are 2 orders of magnitude different.

The data of a fluorescent quenching experiment cannot be fitted correctly by any other combination of constants using different values for Φ_ISC_^0^Φ_RISC_^0^, therefore excluding overparameterization of the three-parameter equation; see Figure 3b. The higher the Φ_ISC_^0^Φ_RISC_^0^, the more *TADF character* the fluorophore possesses. In contrast, if this parameter is equal to 0, we obtain the normal Stern–Volmer equation (**6**), and the fluorophore only emits from the initial singlet state (i.e., it does not demonstrate TADF). Closer to a value of 1, the fluorophore has a rapidly established equilibrium of S_1_ and T_1_ excited states, and the data show a very clear curvature in the Stern–Volmer plot. Indeed, when we compare the curves in Figure 2, the data obtained in toluene have a more pronounced curvature compared to the data in acetonitrile (Φ_ISC_^0^Φ_RISC_^0^ = 0.70 vs 0.29, respectively). Figure 3c further demonstrates the effect of simulating a change in Φ_ISC_^0^ and Φ_RISC_^0^ while keeping *K*_SV_^S^ and *K*_SV_^T^ constant, showing more pronounced curvature at higher values. The limits of eq 10 were further scrutinized by employing the MR-TADF (multiple resonance) fluorophore QAO (also called DiKTa; see Figure 1c),^22,23^ which has a much smaller value for Φ_ISC_^0^Φ_RISC_^0^, limited by the low efficiency of intersystem crossing, Φ_ISC_^0^ = 0.03 (see the Supporting Information).^23^ Even at such low efficiency of TADF, a small amount of curvature was still observed in the Stern–Volmer plots (see the Supporting Information), highlighting the importance of considering both excited states in fluorescence quenching for any photocatalyst displaying TADF.

In conclusion, an extension of the Stern–Volmer equation was derived to describe the nonlinear behavior introduced by the complex excited-state system of TADF photocatalysts that are currently experiencing a rapid growth in their application in photoredox catalysis. This new model comprises three constants: the Stern–Volmer constants for quenching of the singlet and triplet excited states and the quantum yield of successive ISC and RISC. The model was experimentally verified by using three quenchers in four different solvents, showing congruent behavior with rate constants matching those that were independently obtained from time-correlated single photon counting experiments.

## Supporting Information Available

Mathematical derivation of the equations used, general methods, TCSPC data and fits, and additional Stern–Volmer plots and fits (PDF)
Transparent Peer Review report available (PDF)
