# Supplementary material for: An Extension of the Stern–Volmer Equation for Thermally Activated Delayed Fluorescence (TADF) Photocatalysts
Source: J Phys Chem Lett. 2024 Oct 11;15(42):10495–9. doi: 10.1021/acs.jpclett.4c02609 (PMC11548733; doi:10.1021/acs.jpclett.4c02609)
Supplement: Supplementary file 1 — jz4c02609_si_001.pdf [file jz4c02609_si_001.pdf]

Supplementary Information for:

An Extension of the Stern-Volmer Equation for Thermally Activated Delayed Fluorescence (TADF) Photocatalysts

B. Limburg*[1,2]

[1] Secció de Química Orgànica, Facultat de Química, Universitat de Barcelona, Carrer Martí i Franquès 1-11, 08028, Barcelona, Spain
[2] Institut de Química Teòrica i Computacional (IQTC), Carrer Martí i Franquès 1-11, 08028, Barcelona, Spain
* Corresponding author: blimburg@ub.edu

Table of Contents

Mathematical derivation of the main equations 2

General Methods 8

TCSPC Fitting 9

Individual fits for 4CzIPN 12

MR-TADF fluorophore QAO 12

Individual fits for QAO 13

References 14

Mathematical derivation of the main equations

We have a system defined by these states and rate constants:

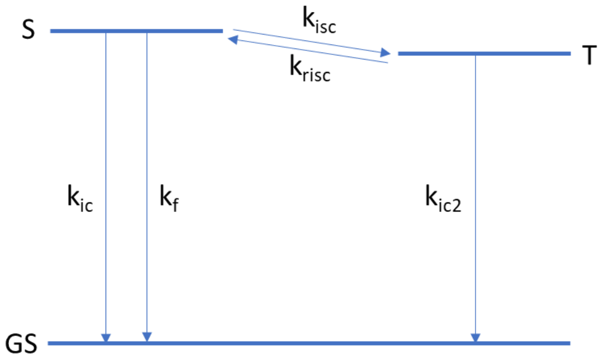

We start at t0 with [GS] = 0, [T] = 0 and [S] = $[S]_0$

We set up the following kinetic differential equations:

$$\frac{d[S]}{dt} = k_{RISC}[T] - k_{ISC}[S] - k_f[S] - k_{ic}[S]$$

$$\frac{d[T]}{dt} = k_{ISC}[S] - k_{RISC}[T] - k_{ic2}[T]$$

Whenever the system decays to GS via the path with rate constant k_f, a photon is emitted that we can measure. Such a system of ordinary differential equations can be solved by putting the system in matrix formulation:

$$\frac{d}{dt}\begin{pmatrix}[S]\\ [T]\end{pmatrix} = \begin{pmatrix}-k_{S_{tot}} & k_{RISC}\\ k_{ISC} & -k_{T_{tot}}\end{pmatrix}\begin{pmatrix}[S]\\ [T]\end{pmatrix}$$

Here $k_{S_{tot}}$ is the sum of all rate constants from the S-state, and $k_{T_{tot}}$ is the sum of all rate constant from the T-state.

We guess that the system can be solved by the following generic equation:

$$\begin{pmatrix}[S]\\ [T]\end{pmatrix} = \begin{pmatrix}v_1\\ v_2\end{pmatrix}e^{\lambda t}$$

And substitute:

$$\lambda\begin{pmatrix}v_1\\ v_2\end{pmatrix}e^{\lambda t} = \begin{pmatrix}-k_{S_{tot}} & k_{RISC}\\ k_{ISC} & -k_{T_{tot}}\end{pmatrix}\begin{pmatrix}v_1\\ v_2\end{pmatrix}e^{\lambda t}$$

And thus

$$\lambda\begin{pmatrix}v_1\\ v_2\end{pmatrix} = \begin{pmatrix}-k_{S_{tot}} & k_{RISC}\\ k_{ISC} & -k_{T_{tot}}\end{pmatrix}\begin{pmatrix}v_1\\ v_2\end{pmatrix}$$

This eigenvalue equation is solved through the characteristic equation:

$$\left|\begin{pmatrix}-k_{S_{tot}} & k_{RISC}\\ k_{ISC} & -k_{T_{tot}}\end{pmatrix} - \lambda \boldsymbol{I}\right| = 0$$

Thus

$$\begin{vmatrix} -k_{S_{tot}} - \lambda & k_{RISC} \\ k_{ISC} & -k_{T_{tot}} - \lambda \end{vmatrix} = 0$$

And therefore

$$\lambda^2 + \lambda\left(k_{S_{tot}} + k_{T_{tot}}\right) + k_{S_{tot}}k_{T_{tot}} - k_{ISC}k_{RISC} = 0$$

To solve for the eigenvalues λ we find:

$$-\lambda_{\pm} = \frac{\left(k_{S_{tot}} + k_{T_{tot}}\right) \pm \sqrt{\left(k_{S_{tot}} + k_{T_{tot}}\right)^2 - 4k_{S_{tot}}k_{T_{tot}} + 4k_{ISC}k_{RISC}}}{2}$$

Which simplifies to

$$-\lambda_{\pm} = \frac{\left(k_{S_{tot}} + k_{T_{tot}}\right) \pm \sqrt{\left(k_{S_{tot}} - k_{T_{tot}}\right)^2 + 4k_{ISC}k_{RISC}}}{2} = \frac{\left(k_{S_{tot}} + k_{T_{tot}}\right) \pm \sqrt{D}}{2}$$

where

$$D = \left(k_{S_{tot}} - k_{T_{tot}}\right)^2 + 4k_{ISC}k_{RISC}$$

The rate constants k_p and k_d are defined as positive numbers (e.g., $k_p = -\lambda_+$):

$$k_{p,d} = \frac{\left(k_{S_{tot}} + k_{T_{tot}}\right) \pm \sqrt{D}}{2}$$

and combinations of the observed rate constants k_p and k_d can be expressed as follows:

$$k_p + k_d = \frac{\left(k_{S_{tot}} + k_{T_{tot}}\right) + \sqrt{D}}{2} + \frac{\left(k_{S_{tot}} + k_{T_{tot}}\right) - \sqrt{D}}{2} = k_{S_{tot}} + k_{T_{tot}}$$

And

$$k_p - k_d = \frac{\left(k_{S_{tot}} + k_{T_{tot}}\right) + \sqrt{D}}{2} - \frac{\left(k_{S_{tot}} + k_{T_{tot}}\right) - \sqrt{D}}{2} = \sqrt{D}$$

Where k_p is the prompt decay (i.e., fast decay, fast rate constants) and k_d is the delayed decay (i.e., slow rate constant). The eigenvectors v_1 and v_2 for k_p and k_d can be calculated from the eigen equation:

$$\begin{pmatrix} -k_{S_{tot}} & k_{RISC} \\ k_{ISC} & -k_{T_{tot}} \end{pmatrix} \begin{pmatrix} v_1 \\ v_2 \end{pmatrix} = -k_{p,d} \begin{pmatrix} v_1 \\ v_2 \end{pmatrix}$$

or

$$\begin{pmatrix} -k_{S_{tot}} + k_{p,d} & k_{RISC} \\ k_{ISC} & -k_{T_{tot}} + k_{p,d} \end{pmatrix} \begin{pmatrix} v_1 \\ v_2 \end{pmatrix} = 0$$

By writing it out as equations for k_p:

$$\left(k_p - k_{S_{tot}}\right)v_1 + k_{RISC}v_2 = 0$$

$$k_{ISC}v_1 + \left(k_p - k_{T_{tot}}\right)v_2 = 0$$

$$v_1 = -\frac{k_{RISC}}{k_p - k_{S_{tot}}} v_2$$

$$v_1 = \frac{k_{RISC}}{\frac{(k_{S_{tot}} + k_{T_{tot}}) + \sqrt{D}}{2} - k_{S_{tot}}} v_2$$

$$v_1 = \frac{2k_{RISC}}{(k_{T_{tot}} - k_{S_{tot}}) + \sqrt{D}} v_2$$

And for k_d

$$v_1 = \frac{2k_{RISC}}{(k_{T_{tot}} - k_{S_{tot}}) - \sqrt{D}} v_2$$

The system therefore decays through a biexponential decay with two constants that describe the initial state (prompt decay), and a second longer-lived decay (delayed decay), and the decay can thus be fitted to the following equation.

$$[S] = Ae^{-k_p t} + Be^{-k_d t}$$

Or

$$\begin{pmatrix} [S] \\ [T] \end{pmatrix} = c_1 \begin{pmatrix} \frac{2k_{RISC}}{(k_{T_{tot}} - k_{S_{tot}}) + \sqrt{D}} \\ 1 \end{pmatrix} e^{-k_p t} + c_2 \begin{pmatrix} \frac{2k_{RISC}}{(k_{T_{tot}} - k_{S_{tot}}) - \sqrt{D}} \\ 1 \end{pmatrix} e^{-k_d t}$$

Under initial conditions, we invoke $[T]_{t=0} = 0$, such that the constant c_1 has to be $-c_2$, and thus that the preexponential factors A and B have the following ratio:

$$\frac{A}{B} = -\frac{2k_{RISC}}{(k_{T_{tot}} - k_{S_{tot}}) + \sqrt{D}} \frac{(k_{T_{tot}} - k_{S_{tot}}) - \sqrt{D}}{2k_{RISC}} = -\frac{(k_{T_{tot}} - k_{S_{tot}}) - \sqrt{D}}{(k_{T_{tot}} - k_{S_{tot}}) + \sqrt{D}}$$

$$= \frac{(k_{S_{tot}} - k_{T_{tot}}) + \sqrt{D}}{(k_{T_{tot}} - k_{S_{tot}}) + \sqrt{D}}$$

Or, alternatively,

$$k_{p,d} = \frac{(k_{S_{tot}} + k_{T_{tot}}) \pm \sqrt{D}}{2}$$

$$k_{p,d} - k_{S_{tot}} = \frac{(k_{T_{tot}} - k_{S_{tot}}) \pm \sqrt{D}}{2}$$

$$\frac{A}{B} = \frac{k_p - k_{T_{tot}}}{k_p - k_{S_{tot}}}$$

$$k_{T_{tot}} = k_p + k_d - k_{S_{tot}}$$

$$k_p - k_{T_{tot}} = k_p - (k_p + k_d - k_{S_{tot}})$$

$$k_p - k_{T_{tot}} = k_{S_{tot}} - k_d$$

$$\frac{A}{B} = \frac{k_{S_{tot}} - k_d}{k_p - k_{S_{tot}}}$$

$$\frac{A}{B}\left(k_p - k_{S_{tot}}\right) = k_{S_{tot}} - k_d$$

$$k_p - k_{S_{tot}} = B\,\frac{k_{S_{tot}} - k_d}{A}$$

$$k_p + B\,\frac{k_d}{A} = B\,\frac{k_{S_{tot}}}{A} + k_{S_{tot}}$$

$$\frac{A}{B}k_p + k_d = \frac{A}{B}k_{S_{tot}} + k_{S_{tot}}$$

$$\frac{A}{B}k_p + k_d = k_{S_{tot}}\left(\frac{A}{B} + \frac{A}{A}\right)$$

$$k_{S_{tot}} = \frac{B}{A+B}\left(\frac{A}{B}k_p + k_d\right)$$

$$k_{S_{tot}} = k_p\,\frac{A}{A+B} + k_d\,\frac{B}{A+B}$$

Further, from the observed decay constants $k_{p,d}$, $k_{S_{tot}}$, and $k_{T_{tot}}$ we can obtain the product $k_{ISC}k_{RISC}$:

$$k_{ISC}k_{RISC} = \frac{\left(k_p - k_d\right)^2 - \left(k_{S_{tot}} - k_{T_{tot}}\right)^2}{4}$$

Remembering that

$$k_p - k_d = \sqrt{D}$$

and

$$D = \left(k_{S_{tot}} - k_{T_{tot}}\right)^2 + 4k_{ISC}k_{RISC}$$

By substituting in the definitions of $k_{S_{tot}}$ and $k_{T_{tot}}$, we can further simplify this as follows:

$$k_{ISC}k_{RISC} = \frac{\left(k_p - k_d\right)^2 - \left(k_p\frac{A}{A+B} + k_d\frac{B}{A+B} - k_p\frac{B}{A+B} + k_d\frac{A}{A+B}\right)^2}{4}$$

$$k_{ISC}k_{RISC} = \frac{\left(k_p - k_d\right)^2 - \left(k_p\left(\frac{A-B}{A+B}\right) + k_d\left(\frac{B-A}{A+B}\right)\right)^2}{4}$$

$$\left(k_p\left(\frac{A-B}{A+B}\right) + k_d\left(\frac{B-A}{A+B}\right)\right)^2 = {k_p}^2\left(\frac{A-B}{A+B}\right)^2 + 2k_p\left(\frac{A-B}{A+B}\right)k_d\left(\frac{B-A}{A+B}\right) + {k_d}^2\left(\frac{B-A}{A+B}\right)^2$$

$$= {k_p}^2\left(\frac{A-B}{A+B}\right)^2 - 2k_pk_d\left(\frac{A-B}{A+B}\right)^2 + {k_d}^2\left(\frac{A-B}{A+B}\right)^2$$

$$= \left(\frac{A-B}{A+B}\right)^2 \left({k_p}^2 - 2k_pk_d + {k_p}^2\right)$$

$$= \left(\frac{A-B}{A+B}\right)^2 \left(k_p - k_d\right)^2$$

$$k_{ISC}k_{RISC} = \frac{\left(k_p - k_d\right)^2 - \left(\frac{A-B}{A+B}\right)^2 \left(k_p - k_d\right)^2}{4}$$

$$k_{ISC}k_{RISC} = \frac{\left(k_p - k_d\right)^2 \left(1 - \left(\frac{A-B}{A+B}\right)^2\right)}{4}$$

$$k_{ISC}k_{RISC} = \frac{\left(k_p - k_d\right)^2 \left(\left(\frac{A+B}{A+B}\right)^2 - \left(\frac{A-B}{A+B}\right)^2\right)}{4}$$

$$k_{ISC}k_{RISC} = \frac{AB}{(A+B)^2}\left(k_p - k_d\right)^2$$

From this product, we can obtain the quantum yield of successive intersystem and reverse intersystem crossings:

$$\frac{k_{ISC}}{k_{S_{tot}}}\frac{k_{RISC}}{k_{T_{tot}}} = \Phi^0_{ISC}\Phi^0_{RISC}$$

This can be expressed in measured constants as follows:

$$\Phi^0_{ISC}\Phi^0_{RISC} = \frac{AB}{\left(Ak_p + Bk_d\right)\left(Bk_p + Ak_d\right)}\left(k_p - k_d\right)^2$$

If we measure the quantum yield of fluorescence (Φ_f), we can deduce the rate of fluorescence, as explained below. The quantum yield can be defined as follows:

$$\Phi^0_f = \Phi^0_{pf}\left(1 + \Phi^0_{ISC}\Phi^0_{RISC} + \left(\Phi^0_{ISC}\Phi^0_{RISC}\right)^2 + \cdots\right)$$

Where the quantum yield of prompt fluorescence (Φ^0_{pf}, i.e., the fluorescence without taking TADF into account) is defined as:

$$\Phi^0_{pf} = \frac{k_f}{k_{S_{tot}}}$$

And therefore, taking the limit of this geometric series, the following equation is produced:

$$\Phi^0_f = \frac{\Phi^0_{pf}}{1 - \Phi^0_{ISC}\Phi^0_{RISC}}$$

From this equation we can obtain k_f.

$$k_f = k_{S_{tot}}\Phi^0_f\left(1 - \Phi^0_{ISC}\Phi^0_{RISC}\right)$$

Fluorescence quenching of TADF-fluorophores

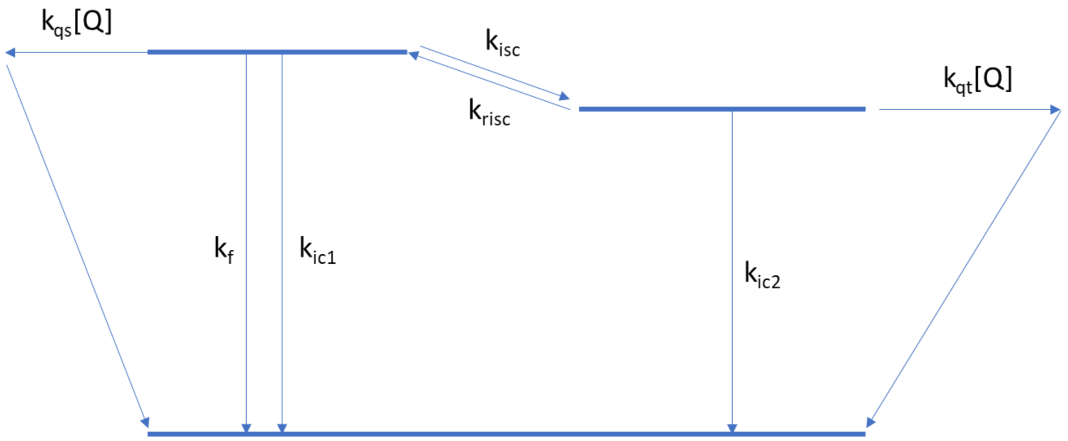

Both the S_1 and the T_1 state can be quenched with bimolecular rate constants k_{qS} and k_{qT}. The quantum yield of fluorescence in the presence of quencher is defined as:

$$\Phi_f(Q) = \frac{\dfrac{k_f}{k_{S_{tot}} + k_{qS}[Q]}}{1 - \dfrac{k_{ISC}k_{RISC}}{\left(k_{S_{tot}} + k_{qS}[Q]\right)\left(k_{T_{tot}} + k_{qT}[Q]\right)}}$$

By analogy with the Stern-Volmer equation, we divide the quantum yield without quencher by that in the presence of quencher:

$$\frac{I_0}{I(Q)} = \frac{\Phi_f^0}{\Phi_f(Q)} = \frac{\dfrac{k_f}{k_{S_{tot}}} \Big/ 1 - \dfrac{k_{ISC}k_{RISC}}{k_{S_{tot}}k_{T_{tot}}}}{\dfrac{k_f}{k_{S_{tot}} + k_{qS}[Q]} \Big/ 1 - \dfrac{k_{ISC}k_{RISC}}{\left(k_{S_{tot}} + k_{qS}[Q]\right)\left(k_{T_{tot}} + k_{qT}[Q]\right)}}$$

$$\frac{\Phi_f^0}{\Phi_f(Q)} = \frac{\dfrac{k_f}{k_{S_{tot}}}\left(1 - \dfrac{k_{ISC}k_{RISC}}{\left(k_{S_{tot}} + k_{qS}[Q]\right)\left(k_{T_{tot}} + k_{qT}[Q]\right)}\right)}{\left(1 - \dfrac{k_{ISC}k_{RISC}}{k_{S_{tot}}k_{T_{tot}}}\right)\left(\dfrac{k_f}{k_{S_{tot}} + k_{qS}[Q]}\right)}$$

$$\frac{\Phi_f^0}{\Phi_f(Q)} = \frac{\dfrac{k_f}{k_{S_{tot}}}\left(1 - \dfrac{k_{ISC}k_{RISC}}{\left(k_{S_{tot}} + k_{qS}[Q]\right)\left(k_{T_{tot}} + k_{qT}[Q]\right)}\right)\dfrac{k_{S_{tot}} + k_{qS}[Q]}{k_f}}{\left(1 - \dfrac{k_{ISC}k_{RISC}}{k_{S_{tot}}k_{T_{tot}}}\right)}$$

$$\frac{\Phi_f^0}{\Phi_f(Q)} = \frac{\frac{k_f}{k_{S_{tot}}}\left(\frac{k_{S_{tot}} + k_{qS}[Q]}{k_f} - \frac{k_{ISC}k_{RISC}}{k_f\left(k_{T_{tot}} + k_{qT}[Q]\right)}\right)}{\left(1 - \frac{k_{ISC}k_{RISC}}{k_{S_{tot}}k_{T_{tot}}}\right)}$$

$$\frac{\Phi_f^0}{\Phi_f(Q)} = \frac{\left(1 + \frac{k_{qS}[Q]}{k_{S_{tot}}} - \frac{k_{ISC}k_{RISC}}{k_{S_{tot}}\left(k_{T_{tot}} + k_{qT}[Q]\right)}\right)}{\left(1 - \frac{k_{ISC}k_{RISC}}{k_{S_{tot}}k_{T_{tot}}}\right)}$$

$$\frac{\Phi_f^0}{\Phi_f(Q)} = \frac{1 + \frac{k_{qS}}{k_{S_{tot}}}[Q] - \frac{k_{ISC}k_{RISC}}{k_{S_{tot}}k_{T_{tot}} + k_{S_{tot}}k_{qT}[Q]}}{1 - \Phi_{ISC}^0\Phi_{RISC}^0}$$

$$\frac{\Phi_f^0}{\Phi_f(Q)} = \frac{1 + \frac{k_{qS}}{k_{S_{tot}}}[Q] - \frac{k_{ISC}k_{RISC}}{k_{S_{tot}}k_{T_{tot}}\left(1 + \frac{k_{qT}}{k_{T_{tot}}}[Q]\right)}}{1 - \Phi_{ISC}^0\Phi_{RISC}^0}$$

$$\frac{\Phi_f^0}{\Phi_f(Q)} = \frac{1 + \frac{k_{qS}}{k_{S_{tot}}}[Q] - \frac{\Phi_{ISC}^0\Phi_{RISC}^0}{1 + \frac{k_{qT}}{k_{T_{tot}}}[Q]}}{1 - \Phi_{ISC}^0\Phi_{RISC}^0}$$

This yields the TADF-corrected Stern-Volmer equation with 3 independent variables:

$$\frac{\Phi_f^0}{\Phi_f(Q)} = \frac{1 + K_{SV}^S[Q] - \frac{\Phi_{ISC}^0\Phi_{RISC}^0}{1 + K_{SV}^T[Q]}}{1 - \Phi_{ISC}^0\Phi_{RISC}^0}$$

Where $K_{SV}^S = \frac{k_{qS}}{k_{S_{tot}}}$ and $K_{SV}^T = \frac{k_{qT}}{k_{T_{tot}}}$ are the Stern Volmer constants.

General Methods

Time-correlated single photon counting (TCSPC) experiments and steady-state fluorimetry were performed on an Edinburgh Instruments FS-5 spectrofluorometer equipped with a SC-20 sample chamber. Samples were degassed through repeated freeze-pump-thaw cycles until no gas bubbles evolved upon thawing and introduced into a 1 cm cuvette fitted with a septum (Hellma 117100F-10-40). Samples were thermostated and stirred at 20 ºC. Excitation was performed at 450 nm or 446 nm using either a Xenon lamp (steady-state fluorimetry) or a laser diode (Edinburgh instruments EPL-450, TCSPC), respectively. Et_3N and DIPEA were added as neat liquids and HEH was added as a saturated solution (1.45 g L^{-1} for toluene, 34.25 g L^{-1} for THF, 58.6 g L^{-1} for DMF, and 4.29 g L^{-1} for MeCN) using precision syringes. Data were fitted and plotted

using in-house written python scripts. 4CzIPN was synthesized according to a reported procedure.[1] QAO was synthesized according to a reported procedure.[2] All solvents and reagents were obtained from commercial sources and used without further purification.

TCSPC Fitting

Obtaining the correct fitted values from TCSPC of TADF fluorophores can be difficult due to the large differences in the rate of prompt and delayed fluorescence. For example, the TCSPC of 4CzIPN in toluene using 1024 channels only has a few points on the prompt decay when taking into account the full decay, whereas measuring the same at a shorter time-window does not show the full delayed fluorescence decay (see Figure S1).

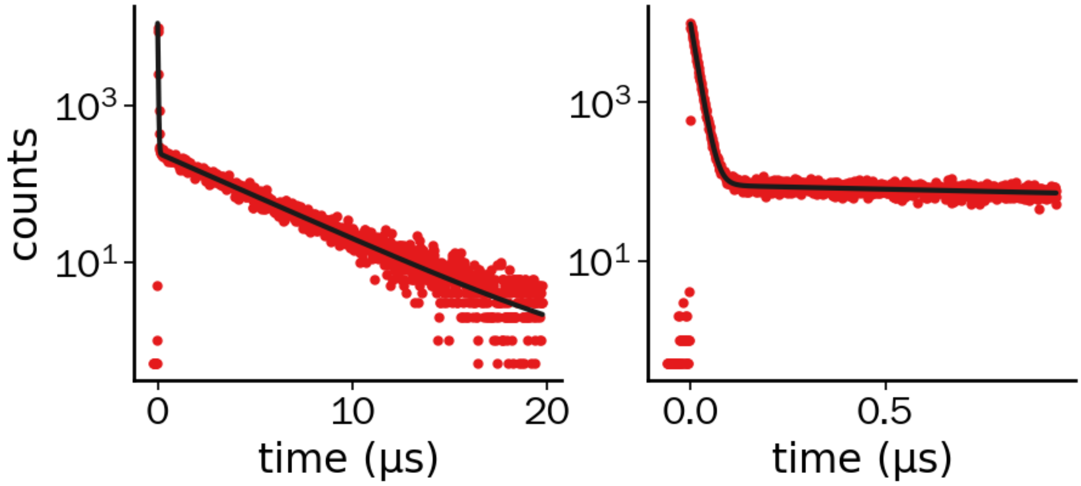

Figure S1. TCSPC of 4CzIPN (50 μM) in toluene at 20 ºC, λ_{ex} = 446 nm. Left: 20 μs window, right: 1 μs window.

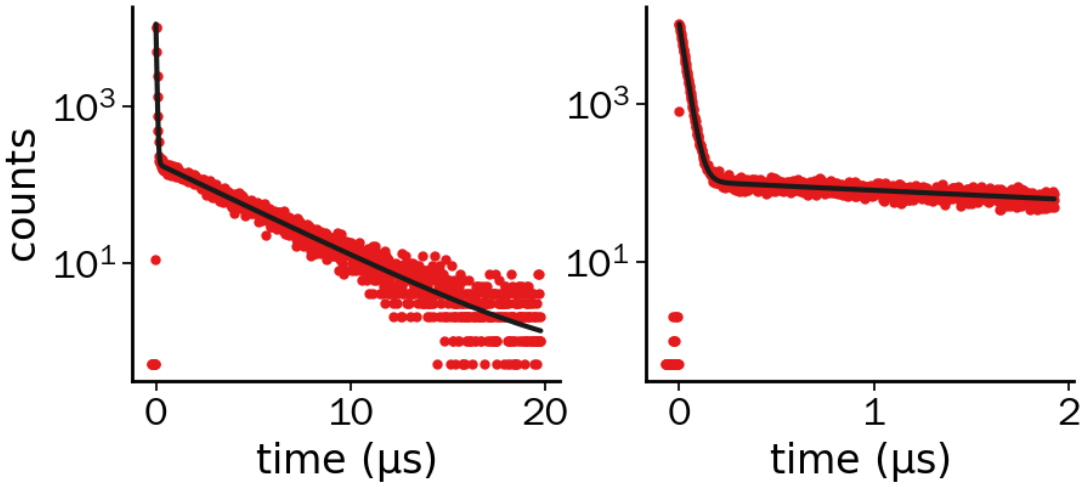

Figure S2. TCSPC of 4CzIPN (50 μM) in THF at 20 ºC, λ_{ex} = 446 nm. Left: 20 μs window, right: 2 μs window.

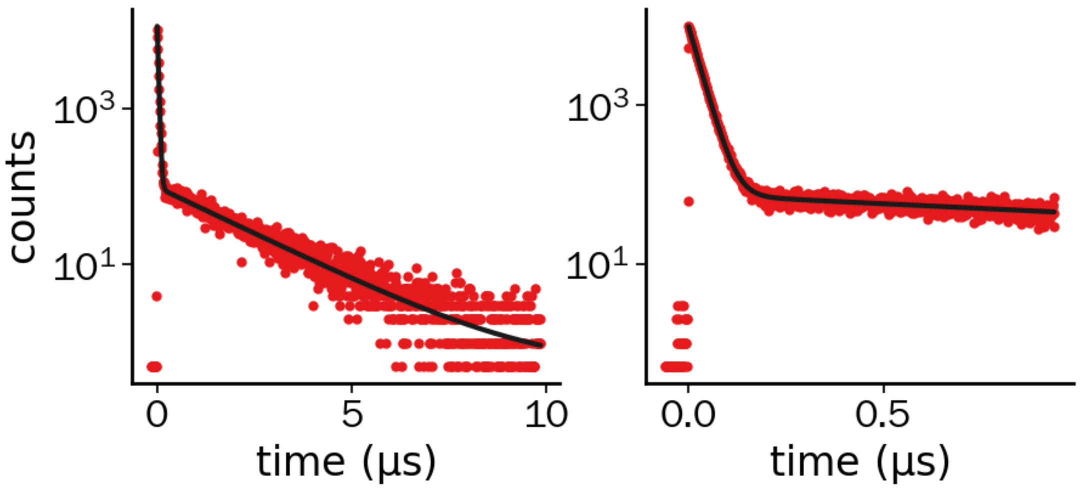

Figure S3. TCSPC of 4CzIPN (50 μM) in DMF at 20 ºC, λ_{ex} = 446 nm. Left: 10 μs window, right: 1 μs window.

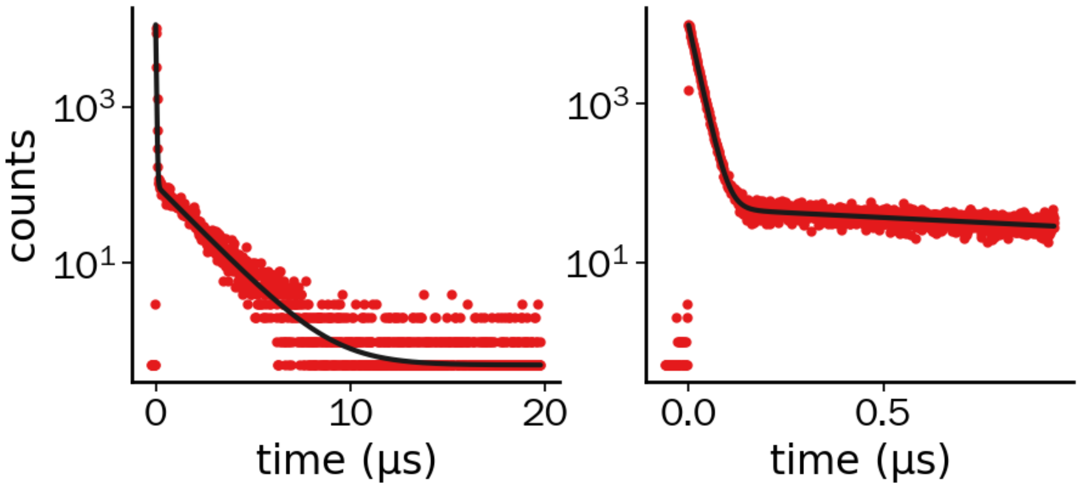

Figure S4. TCSPC of 4CzIPN (50 μM) in MeCN at 20 ºC, λ_{ex} = 446 nm. Left: 20 μs window, right: 1 μs window.

The fit of former, therefore, is imprecise for values of A and k_p because of a lack of data on the prompt decay. In contrast, the delayed fluorescence rate constant k_d can be determined precisely (to 0.9% error, see Table S1). Therefore, in order to determine both the prompt and the fast components with good precision, first the rate constant of delayed fluorescence k_d is determined using a wider range of 20 μs, and then the other 3 constants can be determined by fitting the smaller range, while keeping k_d fixed. As can be seen from the data in Table S1, the standard errors on the fitting constants decreases significantly when following this procedure, which therefore increases the accuracy of any calculations done with these constants.

The TCSPC experiments and fits for the other solvents (THF, DMF and MeCN) are displayed in Figures S2, S3 and S4, and fitted constants are shown in Tables S2, S3 and S4.

Table S1. Fitting constants for the data in Figure S1.

	20 µs window	1 µs window
A	$1.10 \cdot 10^4 \pm 0.34 \cdot 10^4$ (± 31%)	$9.72 \cdot 10^3 \pm 0.96 \cdot 10^3$ (± 9.8%)
k_p	$4.66 \cdot 10^7 \pm 0.71 \cdot 10^7$ (± 15%)	$6.75 \cdot 10^7 \pm 0.26 \cdot 10^7$ (± 3.9%)
B	247 ± 6.2 (± 2.5%)	90.3 ± 1.3 (± 1.4%)
k_d	$2.54 \cdot 10^5 \pm 0.023 \cdot 10^5$ (± 0.9%)	$2.54 \cdot 10^5$ (fixed)

Table S2. Fitting constants for the data in Figure S2.

	20 µs window	2 µs window
A	$1.10 \cdot 10^4$	$9.97 \cdot 10^3 \pm 1.03 \cdot 10^3$ (± 10%)
k_p	$2.97 \cdot 10^7 \pm 0.40 \cdot 10^7$ (± 13%)	$3.69 \cdot 10^7 \pm 0.15 \cdot 10^7$ (± 4.1%)
B	186 ± 5.5 (± 3.0%)	104 ± 1.4 (± 1.3%)
k_d	$2.73 \cdot 10^5 \pm 0.028 \cdot 10^5$ (± 1.0%)	$2.73 \cdot 10^5$ (fixed)

Table S3. Fitting constants for the data in Figure S3.

	10 µs window	1 µs window
A	$1.10 \cdot 10^4 \pm 0.0088 \cdot 10^4$ (± 0.8%)	$9.87 \cdot 10^3 \pm 0.682 \cdot 10^3$ (± 6.9%)
k_p	$3.87 \cdot 10^7 \pm 0.42 \cdot 10^7$ (± 11%)	$3.99 \cdot 10^7 \pm 0.10 \cdot 10^7$ (± 2.5%)
B	97.2 ± 3.64 (± 3.7%)	74.5 ± 1.04 (± 1.4%)
k_d	$5.49 \cdot 10^5 \pm 0.076 \cdot 10^5$ (± 1.4%)	$5.49 \cdot 10^5$ (fixed)

Table S4. Fitting constants for the data in Figure S4.

	20 µs window	1 µs window
A	$1.10 \cdot 10^4 \pm 0.42 \cdot 10^4$ (± 38%)	$9.85 \cdot 10^3 \pm 0.85 \cdot 10^3$ (± 8.6%)
k_p	$4.01 \cdot 10^7 \pm 0.61 \cdot 10^7$ (± 15%)	$5.15 \cdot 10^7 \pm 0.15 \cdot 10^7$ (± 2.9%)
B	100 ± 5.2 (± 5.2%)	48.2 ± 0.75 (± 1.6%)
k_d	$5.78 \cdot 10^5 \pm 0.103 \cdot 10^5$ (± 1.8%)	$5.78 \cdot 10^5$ (fixed)

Individual fits for 4CzIPN

The fits in the main text were performed globally per solvent to obtain a more precise value for $\Phi_{ISC}^{0}\Phi_{RISC}^{0}$. For completeness, the fits for each quencher individually (i.e., not sharing $\Phi_{ISC}^{0}\Phi_{RISC}^{0}$ for the fit between experiments of different quenchers) are shown in Figure S5.

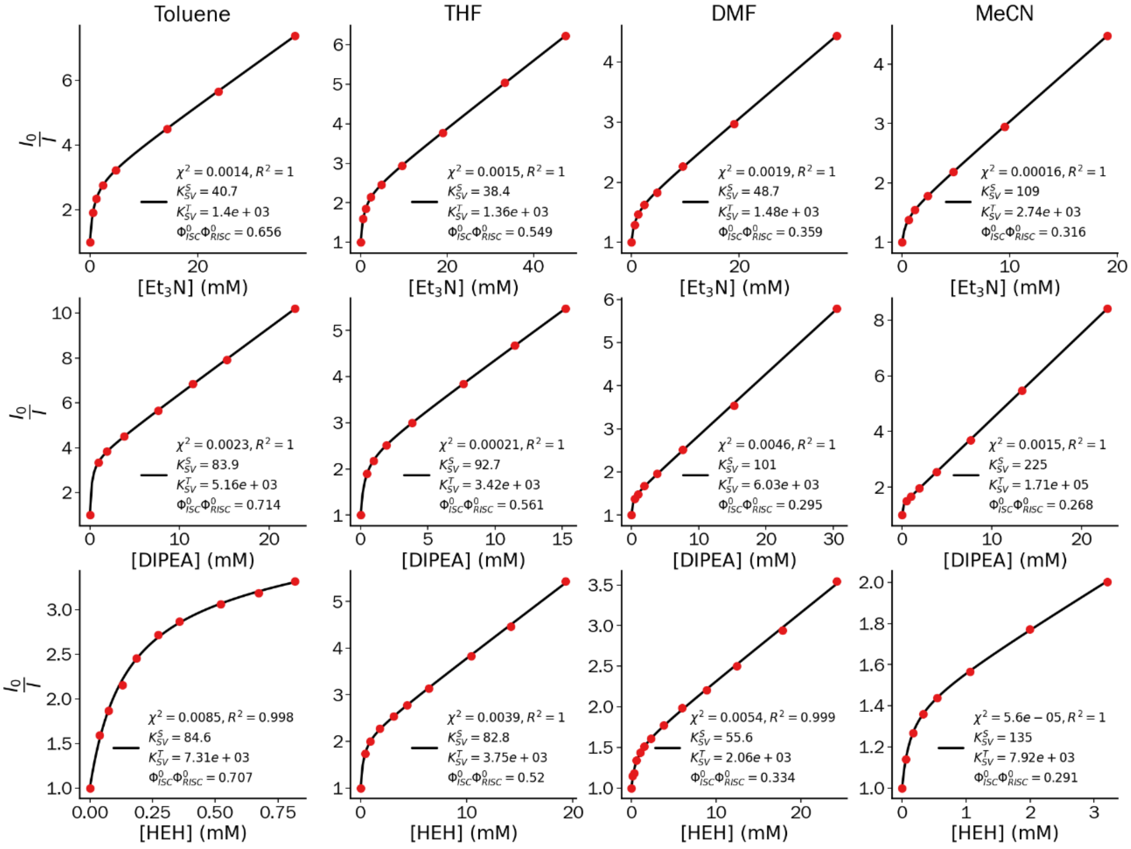

Figure S5. Stern-Volmer plots of TADF-photocatalyst 4CzIPN (50 µM) in four solvents (toluene, THF, DMF and MeCN) with three different quenchers (Et_3N, DIPEA, HEH) thermostated at 20 ºC, λ_{ex} = 450 nm. Individual fits to equation 10 shown as the black continuous line, and goodness of fit χ^2 and R^2indicated for each fit.

MR-TADF fluorophore QAO

Fluorescence quenching experiments were conducted with QAO as fluorophore (see Figure S6). This dye has a low quantum yield of intersystem crossing (Φ_{ISC}^{0} = 0.03),[2] and our equipment did not allow for the measurement of the TADF by TCSPC. However, the quenching experiments did show a minor amount of curvature at low concentrations of quenchers, but precise determination of constants was difficult due to the high amount of experimental error compared to the small amount of quenching at these concentrations, due to the low value of Φ_{ISC}^{0}.

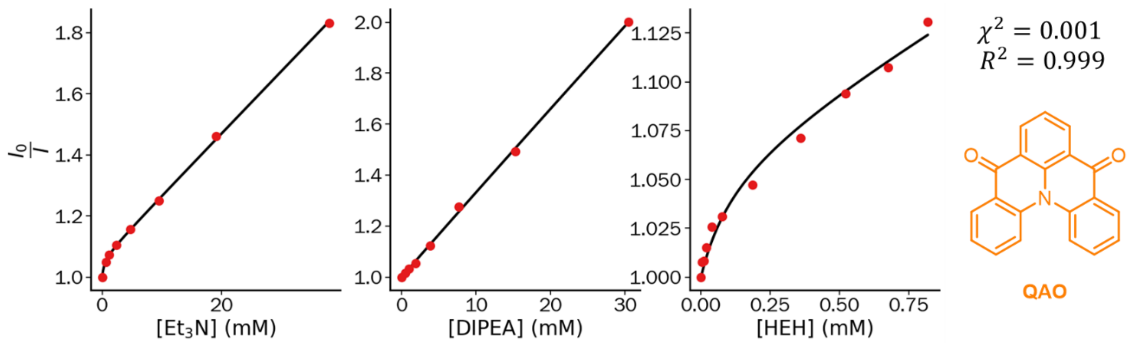

Figure S6. Stern-Volmer plots of TADF-photocatalyst QAO (5 µM) in toluene with three different quenchers (Et_3N, DIPEA, HEH) thermostated at 20 ºC, λ_{ex} = 433 nm (red circles) and global fit of all data to equation **10** (black continuous lines).

Table S5. Obtained fitting constants from fluorescence quenching experiments in Figure S6. [a]

Solvent	Quencher	K_{SV}^{S} (M^{-1}) [b]	$K_{SV}^{T}\times 10^3$ (M^{-1}) [b]	$\Phi_{ISC}^{0}\Phi_{RISC}^{0}$ [b]
Toluene	Et_3N	19.1 ± 0.42	2.43 ± 1.41	0.06 ± 0.007 (0.03) [c]
	DIPEA	30.0 ± 0.69	0.026 ± 0.027 [b]	
	HEH	79.0 ± 10.5	7.93 ± 2.78	

[a] Uncertainties are the standard error of the fitted constants. [b] Curvature obscured by experimental error, therefore not allowing the determination of K_{SV}^{T}. [c] From reference 2, assuming that Φ_{RISC}^{0} = 1.

Individual fits for QAO

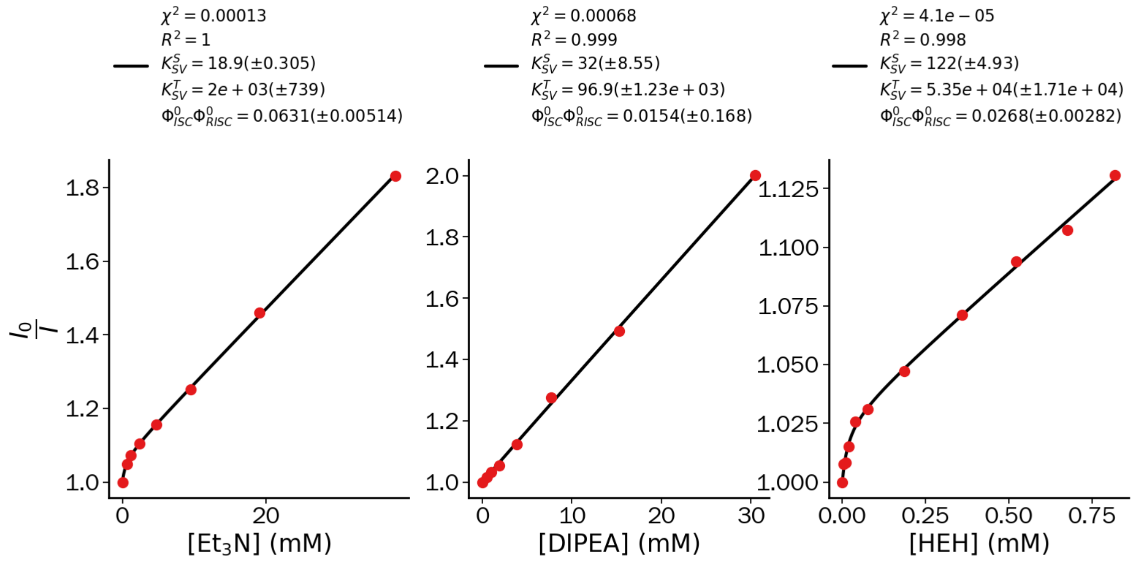

Figure S7. Stern-Volmer plots of TADF-photocatalyst QAO (5 µM) in toluene with three different quenchers (Et_3N, DIPEA, HEH) thermostated at 20 ºC, λ_{ex} = 433 nm. Individual fits to equation 10 shown as the black continuous line, and goodness of fit χ^2 and R^2 indicated for each fit.

References

(1) Uoyama, H.; Goushi, K.; Shizu, K.; Nomura, H.; Adachi, C. Highly Efficient Organic Light-Emitting Diodes from Delayed Fluorescence. *Nature* **2012**, *492*, 234–238.

(2) Hall, D., Suresh, S. M., dos Santos, P. L., Duda, E., Bagnich, S., Pershin, A., Rajamalli, P., Cordes, D. B., Slawin, A. M. Z., Beljonne, D., Köhler, A., Samuel, I. D. W., Olivier, Y., & Zysman-Colman, E. Improving Processability and Efficiency of Resonant TADF Emitters: A Design Strategy. *Adv. Optical Mater.* **2020**, *8*, 1901627.
