# Supplementary material for: An Extension of the Stern–Volmer Equation for Thermally Activated Delayed Fluorescence (TADF) Photocatalysts
Source: J Phys Chem Lett. 2024 Oct 11;15(42):10495–9. doi: 10.1021/acs.jpclett.4c02609 (PMC11548733; doi:10.1021/acs.jpclett.4c02609)
Supplement: Supplementary file 2 — jz4c02609_si_002.pdf [file jz4c02609_si_002.pdf]

jz-2024-026094.R1

Name: Peer Review Information for "An Extension of the Stern-Volmer Equation for Thermally Activated Delayed Fluorescence (TADF) Photocatalysts"

First Round of Reviewer Comments

Reviewer: 1

Comments to the Author

The manuscript by Limburg presents a calibrated Stern-Volmer equations used for elucidating excited quenching behavior in TADF emitters. Based on the deduced the equations, the highly related fitting was demonstrated. This provides a valuable method for calculating the quenching parameters and excited state transition rates. I am very satisfised with this work and would recommend the manuscript for publication in J. Phys. Chem. Lett. after addressing following minor revisions.

1. The author investigated quenching behavior in three different solvents to validate the proposed equations. However, to further demonstrate the generality of the calibrated Stern-Volmer equation, it would be beneficial to test it in a more polar solvent such as DMF.

2. The R-squared fitting values should be included in Figures 2 and 3 for clarity and completeness

Reviewer: 2

Comments to the Author

This manuscript derives an extension of the standard Stern-Volmer equation which takes into account quenching in both the singlet and triplet excited states. Experimental quenching studies with 4CzIPN in a series of solvents and quenchers demonstrate the validity of this extension and allow for the determination of quantum yields for (reverse) intersystem crossing. I find this work substantial and support its publication in The Journal of Physical Chemistry Letters with the following comments.

1. The utility of this extension would be better justified if the authors conducted quenching experiments with other TADF dyes. Perhaps an MR-TADF emitter (e.g., QAO - also commonly used in photocatalysis; other dyes are also possible)

2. Reports by Zhang, Mei, and You (Angew. Chem. Int. Ed. 2020, 59, 18062), (Org. Lett. 2020, 22, 9699) show linear SV plots when quenching 4CzIPN. Can the author clarify the discrepancy between reported behaviour and their own?

3. The author should clarify the term “traditional organic dyes” as there are numerous organic photocatalysts which feature purely fluorescent excited states.

Author's Response to Peer Review Comments:

Dear Editor,

Many thanks for your consideration and the opportunity to better my manuscript entitled "An Extension of the Stern-Volmer Equation for Thermally Activated Delayed Fluorescence (TADF) Photocatalysts". I believe to have fully addressed the reviewer's comments, and hope they, and you, will be convinced by the clarifications in the text, as well as the additional experiments performed.

I attach the revised manuscript, revised supporting information, manuscript with changes highlighted, and answers to the reviewer's comments.

Thank you in advance for considering my revised manuscript.

Best wishes,

Bart Limburg

**Answers to the reviewer comments:**

Reviewer 1:

The manuscript by Limburg presents a calibrated Stern-Volmer equations used for elucidating excited quenching behavior in TADF emitters. Based on the deduced the equations, the highly related fitting was demonstrated. This provides a valuable method for calculating the quenching parameters and excited state transition rates. I am very satisfised with this work and would recommend the manuscript for publication in J. Phys. Chem. Lett. after addressing following minor revisions.

I thank the reviewer for their kind comments and their suggestions which I address below.

1. The author investigated quenching behavior in three different solvents to validate the proposed equations. However, to further demonstrate the generality of the calibrated Stern-Volmer equation, it would be beneficial to test it in a more polar solvent such as DMF.

According to the reviewer's comment, the quenching experiments have been performed as well in DMF and added to the manuscript.

2. The R-squared fitting values should be included in Figures 2 and 3 for clarity and completeness

The R-squared values (all > 0.999) have been added to the manuscript. Due to the function being non-linear, the R-squared value is generally considered not an adequate measurement for the goodness-of-fit. Therefore, in addition to the R-squared value, I have added the Chi-squared values as well.

Reviewer: 2

This manuscript derives an extension of the standard Stern-Volmer equation which takes into account quenching in both the singlet and triplet excited states. Experimental quenching studies with 4CzIPN in a series of solvents and quenchers demonstrate the validity of this extension and allow for the determination of quantum yields for (reverse) intersystem crossing. I find this work substantial and support its publication in The Journal of Physical Chemistry Letters with the following comments.

I thank the reviewer for their kind comments, and for their excellent points to improve the manuscript, which I address below.

1. The utility of this extension would be better justified if the authors conducted quenching experiments with other TADF dyes. Perhaps an MR-TADF emitter (e.g., QAO - also commonly used in photocatalysis; other dyes are also possible)

I have synthesized and employed QAO as an additional dye in addition to 4CzIPN. Generally, these types of dyes have low intersystem crossing quantum yields, which makes the described curvature much less pronounced. However, as I now show in the SI, the effect is still present and important to take into account.

2. Reports by Zhang, Mei, and You (Angew. Chem. Int. Ed. 2020, 59, 18062), (Org. Lett. 2020, 22, 9699) show linear SV plots when quenching 4CzIPN. Can the author clarify the discrepancy between reported behaviour and their own?

There are several factors that can lead to an apparent linear function even though there should be a curved feature. First and foremost, experimental errors on the datapoints might lead to the curvature being obscured by the deviation of the data-points. Furthermore, as I now more clearly mention in the manuscript, the Stern-Volmer plot might appear linear if the first datapoint is obtained at a quencher concentration where the triplet state is (nearly) fully quenched. This,

however, leads to a Stern-Volmer plot where the y-axis intersect is not at 1 (Figure 3a, added to the new version of the manuscript, demonstrates this). The fit when using the normal Stern-Volmer equation should always pass through y(0)=1, since otherwise the slope does not have a clear physical meaning. Last, insufficient exclusion of dioxygen can lead to nearly quantitative triplet quenching, which would again lead to a linear plot. These points have been clarified in the manuscript.

3. The author should clarify the term “traditional organic dyes” as there are numerous organic photocatalysts which feature purely fluorescent excited states.

I have removed the term “traditional organic dyes” from the manuscript, and replaced it with the term non-TADF dyes to avoid confusion.
